# TIMAP downregulation in Burkitt’s lymphoma reveals key molecules and signaling pathways in B-cell lymphomagenesis

**DOI:** 10.3389/ebm.2025.10533

**Published:** 2025-10-15

**Authors:** Marya Obeidat, Saleh Tadros, Batool Ismail, Ayah Al-Khaldi

**Affiliations:** Department of Medical Laboratory Sciences, Faculty of Applied Medical Sciences, Jordan University of Science and Technology, Irbid, Jordan

**Keywords:** TIMAP, PPP1R16B, RNA sequencing, B-cell lymphoma, transcriptome

## Abstract

Burkitt’s lymphoma (BL) is an aggressive subtype of B-cell non-Hodgkin’s lymphoma, known for its rapid tumor growth and poor prognosis. Transforming growth factor beta-inhibited membrane-associated protein (TIMAP) is a regulatory subunit of protein phosphatase 1 catalytic subunit, enriched in lymphoid tissues, and upregulated in various cancers. Despite suggestions that TIMAP promotes lymphomagenesis in a *c-myc*-driven model, its precise role remains unclear. This study aimed to investigate the contribution of TIMAP to B-cell lymphomagenesis by examining transcriptomic changes upon TIMAP downregulation in BL cells. Raji BL cells were transfected with 2′Fluoro Arabinonucleic acid (FANA)-antisense oligonucleotides (ASO) targeting TIMAP (FANA-ASO-TIMAP) or a scramble control (FANA-ASO-Scramble). TIMAP expression was significantly reduced at the mRNA (0.70 ± 0.04, p = 0.001) and protein levels (median = 0.73, IQR = 0.13, p = 0.002). RNA sequencing identified 2,368 differentially expressed genes (DEGs), of which 1,326 were upregulated, and 1,042 were downregulated. Gene Ontology analysis revealed that the DEGs were primarily involved in cellular processes, DNA replication, intracellular signal transduction, and apoptosis. Pathways related to lymphoma progression, such as B-cell receptor signaling, p53 signaling, and mTOR signaling, were notably affected. Key genes such as *PAK3*, *LINC00487*, *AID*, *PURPL,* and *BCL2* were among the most dysregulated, highlighting TIMAP’s role in critical oncogenic pathways in B-cell Lymphoma. These findings suggest that TIMAP is a key regulator of gene expression and signaling pathways in B-cell lymphomagenesis and could serve as a potential therapeutic target for novel treatments.

## Impact statement

This manuscript contributes to the growing body of research on Burkitt's lymphoma (BL) by investigating the role of TIMAP, a protein implicated in cancer progression. The study reveals that even a partial reduction in TIMAP expression causes significant changes in the behavior of BL cells, particularly in genes and pathways linked to cell survival, proliferation, and apoptosis. Through transcriptomic analysis, the research identifies over 2,300 differentially expressed genes and highlights the disruption of critical signaling pathways like B-cell receptor signaling and mTOR, essential in lymphoma development. These findings deepen our understanding of how TIMAP regulates key processes in B-cell lymphomagenesis and suggest that TIMAP could be a promising target for new lymphoma therapies. By positioning TIMAP as a central player in lymphoma biology, the study opens new avenues for targeted treatments and offers insights into the disease's molecular mechanisms. Future research can further explore TIMAP’s therapeutic potential in clinical applications.

## Introduction

Blood cancers, which account for 6% of all malignancies [1], are a group of neoplastic illnesses that primarily involve bone marrow, blood, and lymphatic tissue [2]. Based on the site of involvement, hematological malignancies are divided into leukemia, lymphoma, and myeloma [3]. Lymphoma is caused by the abnormal proliferation of blood lymphocytes (B, T, and Natural Killer (NK) cells) at various stages of maturation [4] and accounts for 5% of all cancer cases [5]. These malignant cells accumulate in the lymphatic system (lymph nodes, spleen, thymus, and bone marrow) and other parts of the body [3]. Hodgkin Lymphoma (HL) and Non-Hodgkin Lymphoma (NHL) are two subtypes of lymphoma distinguished by the presence of Reed-Sternberg cells in the biopsies of HL patients [6]. Approximately 90% of lymphoma cases are NHL, which is more common among men than women [7]. NHL is further subdivided into subgroups based on the kind of malignant lymphocyte (B-cells, T-cells, or natural killer (NK)-cells), clinical presentation, aggressiveness, prognosis, and treatment response [8]. Most cases of NHL are B-cell lymphomas, which are further divided into indolent (low-grade), such as follicular lymphoma (FL), and aggressive (high-grade), such as diffuse large B-cell lymphoma (DLBCL) and Burkitt’s Lymphoma (BL).

Burkitt’s lymphoma (BL) is an aggressive B-cell subtype of NHL that often affects children and, to a lesser extent, young individuals in malaria-endemic areas [9]. BL is distinguished by rapid cell division, as seen by cell-cycle markers like Ki-67 (>95% of cells are positive) [10]. Additionally, it is one of the neoplasms that has been connected to Epstein-Barr Virus (EBV), Human Immunodeficiency Virus (HIV), and chromosomal translocations that lead to overexpression of *c-Myc* oncogene [11–13]. While *c-Myc* overexpression enhances B-cell proliferation, it also promotes cell death [14]. As a result, lymphoma development requires extra genes that support cell survival.

The World Health Organization (WHO) classifies BL into three types: endemic, sporadic, and immunodeficiency-related [15], all have the same morphology, genetic features, and immunostaining results. Physical examination, laboratory tests (Complete Blood Count (CBC), Blood Film Examination (BFE), and assessment of bone marrow and lymph node biopsies), radiography, and cytogenetic analysis are all required for a clear diagnosis of BL [10]. Treatment must begin as soon as a diagnosis is made since BL is fatal if left untreated. BL is often treated with chemotherapeutic and immune-targeted medicines for months [16]. Even though BL is susceptible to chemotherapy [16, 17], chemotherapy-related toxicity and infections can develop, especially in immunocompromised patients [18]. Targeting molecules involved in BL pathogenesis as a treatment option against BL reduces non-specific damage to normal cells and minimizes side effects from conventional therapies [19].

TIMAP (Transforming Growth Factor Beta 1 (TGF-ß1) Inhibited Membrane-Associated Protein) is a member of the Myosin Phosphatase Targeting subunits (MYPT) family that forms a holoenzyme complex with Ser/Thr Protein Phosphatase 1 catalytic subunit (PP1c) to regulate its substrate specificity, activity, and localization [20]. TIMAP is predominantly expressed in endothelial cells, white blood cells (B, T, NK, and Dendritic cells), and several tissues, including the central nervous system (CNS), bone marrow, and lymphoid organs [21–23]. TIMAP has been reported to be upregulated in a variety of solid cancers [23], including breast cancer [24] and head and neck cancer [23]. *TIMAP* transcript was identified among the upregulated genes in diffuse DLBCL and peripheral T-cell lymphoma not otherwise specified (PTCL-NOS) [25], BL cell lines, and leukemia cell lines [23]. It is a prognostic biomarker in HER-2-negative breast cancer [24], head and neck cancer [23, 26], liver cancer, renal cancer [23], and glioblastoma multiform [27]. Furthermore, a large-scale study in a c*-Myc* mice lymphoma model sensitive to apoptosis found two deregulated oncogenes, TIMAP and histone deacetylase isoform 6 (HDAC6), demonstrating their significance in lymphomagenesis [28]. Nonetheless, to date, the functional role of TIMAP in lymphoma is underexplored.

Numerous protein partners for the TIMAP-PP1c complex have been identified, mainly in studies on endothelial cells (EC), which are involved in pathways that regulate cell growth, adhesion, and migration [20]. Among TIMAP partners in EC is a small nuclear ribonucleoprotein U5 (U5 snRNP) that is involved in RNA splicing [29]. Additionally, a recent study in neuroblastoma cells found multiple nuclear protein partners for TIMAP, including splicing factor proline- and glutamine-rich (SFPQ) proteins and heterogeneous nuclear ribonucleoprotein A1 (hnRNPA1) [27]. These findings strongly suggest that TIMAP may play a role in the regulation of gene transcription.

Cumulative evidence strongly indicates the role of TIMAP in cell transformation, likely through transcriptional regulation. Despite this, studies explaining how TIMAP works in malignant cells and identifying its target genes are still lacking. Since TIMAP is implicit in lymphomagenesis and its expression is upregulated in various lymphoma cell lines [23]. We sought to identify transcripts whose expression is deregulated following TIMAP knockdown in BL cells, to gain insight into the cellular pathways that might be influenced by TIMAP expression. Ultimately, these findings will help researchers understand the pathogenic role of TIMAP in lymphomagenesis, particularly in BL.

## Materials and methods

### Cell lines and cell culture

Raji (ATCC CCL-86^™^) and Daudi (ATCC CCL-213^™^) BL cell lines were purchased from the American Type Culture Collection (ATCC) and cultured in sterile RPMI 1640 w/L-Glutamine (Euro Clone, Cat. No. ECB 2000L) supplemented with 10% heat-inactivated fetal bovine serum (FBS) (PAN BIOTECH, Cat. No. P30-3306 and Gibco, Cat. No.10500-064) and 1% penicillin/streptomycin (Euro Clone, Cat. No. ECB 3001D). Cell cultures were maintained at 37 °C in a humidified 5% CO2 incubator and used at passages 4–7. Cell growth and morphology were monitored daily using an inverted microscope, and the growth media were replaced every 2-3 days or as needed.

### RNA extraction

Total RNA was extracted from cells using Qiagen RNeasy^®^ Micro kit (Cat. No. 74004) as directed by the manufacturer. A Thermo Fisher ND-2000 nanodrop^™^ spectrophotometer was used to determine the quality of extracted RNA. RNA samples were stored at −80 °C for further analysis.

### Reverse transcriptase-polymerase chain reaction (RT-PCR)

A total mass of 500 µg RNA was reverse transcribed into cDNA using a two-step QuantiTect^®^ Reverse Transcription Kit (Qiagen, Cat. No. 205311). The PCR mixture was prepared of 2 µL 5x HOT FIREPol Blend Master Mix (Solis BioDyne, Cat. No. 04-25-00S25), 0.5 µL forward (F) and reverse (R) primers (Table 2), 1 µL of cDNA template, and 6 µL of nuclease-free water. The PCR cycles were as follows: 95 °C for 12 min, followed by 35 cycles of 95 °C for 30 s, 61 °C for 30 s, 72 °C for 2 min, and 72 °C for 10 min. Using a UV transilluminator, the PCR products were visualized on a 2% agarose gel.

### Immunofluorescence

The expression of TIMAP protein was examined in Raji and Daudi BL cells seeded on glass coverslips in a 24-well plate containing 1 mL complete growth media/well for 24 h at 37 °C in a humidified 5% CO2 incubator. Afterward, the plate was centrifuged at 250 × g for 7 min at room temperature (RT), and cells were rinsed with 1x phosphate-buffered saline (PBS) and fixed in 10% formalin for 15 min at RT. The cells were then permeabilized with 0.1% Triton X 100 in PBS for 15 min in the dark, blocked with 1 mL of 2% bovine serum albumin (BSA) in PBS for 1 h at RT on a shaker to block non-specific binding, and incubated overnight at 4 °C with 1: 500 primary rabbit polyclonal anti-PPP1R16B antibody (MyBioSource, Cat. No. MBS417306) in 1% BSA. The following day, the plate was further incubated for 20 min on ice on a shaker, followed by three washes with PBS for 5 min. Then 500 µL of secondary goat anti-rabbit antibody (Alexa Fluor^®^ 488) (Abcam Cat# ab150077, RRID:AB_2630356) diluted at 1:1000 in 1% BSA was added and incubated for 40 min on a shaker in the dark. Finally, the plate was rinsed three times with PBS, and coverslips were placed on a drop of mounting media with DAPI counterstain (Abcam, Cat. No. ab104139) on frosted glass slides and sealed with nail polish to avoid drying. The Nikon Eclipse E600 microscope was used to capture images at ×100 magnification.

### Immunohistochemistry (IHC)

Archived paraffin-embedded lymph node tissue samples from a healthy control, a BL patient, a DLBCL patient, and an FL patient were sectioned to a thickness of 4 μm and mounted on Superfrost Plus glass slides for IHC processing using the BenchMark ULTRA system (Roche Diagnostics, Risch-Rotkreuz, Switzerland). To assess TIMAP protein expression, an anti-PPP1R16 B rabbit polyclonal antibody (MyBioSource, Inc, San Diego, United States) was used at a 1:400 dilution [24, 26].

### TIMAP knockdown

Four different constructs of 2′-Deoxy-2′-fluoro-arabinoguanosine-Antisense Oligonucleotides (FANA-ASOs) targeting TIMAP mRNA [FANA-ASO-TIMAP (AUM*silence* ^*TM*^ ASO)], and a scramble negative control [FANA-ASO-scramble (AUM*scr* ^*TM*^ ASO)] were purchased from AUM Bio Tech, LLC (PA, United States) (Table 1). FANA ASOs are 2′-deoxy-2′-fluoroarabinonucleotides that mimic DNA [30]. These ASOs form FANA: RNA hybrids, like native DNA: RNA hybrids, and can trigger RNase H-mediated RNA cleavage. Due to their chemical modifications, FANA ASOs are self-delivered into cells, including hard-to-transfect cells [31], without a need for transfection reagents, reducing cell toxicity and enabling targeted mRNA degradation. Raji cells were plated in complete growth media at a density of 50% in 12-well or 24-well plates as directed by the manufacturer. Afterward, the cells were gently mixed with 2 µM of FANA-ASO diluted in the growth media, and the growth media was refreshed 48 h after treatment. A soup of 4 FANA-ASO-TIMAP constructs was used to knock down TIMAP. RNA and protein extractions were conducted 72 h after treatment.

**TABLE 1:** AUM*silence* ^*TM*^ sequences and their target regions on the TIMAP transcript.

AUM*silence* ASOs	AUM*silence* ^*TM*^ sequence (5′- 3′)	Target region in TIMAP transcript (NM_015568.4)
AUM*scr* ^*TM*^	CCT​TCC​CTG​AAG​GTT​CCT​CC	No target
AUM*silence* ^*TM*^1	AAT​ATA​CCG​AGG​TCC​CAT​TGC	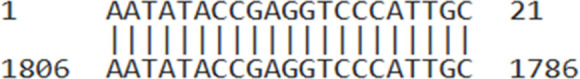
AUM*silence* ^*TM*^2	ACC​TAA​CGT​AGA​GGC​TGG​CAT	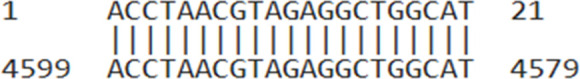
AUM*silence* ^*TM*^3	GAG​ACT​AGG​AGA​TAC​GGC​AAC	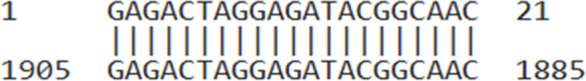
AUM*silence* ^*TM*^4	TAG​ATC​ATC​CTG​TCC​TGT​TCC	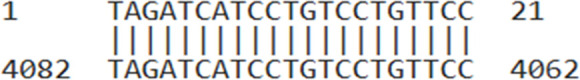

In each experiment, equal numbers of cells were seeded following viable cell counting by trypan blue exclusion using a hemocytometer [32]. This method involves mixing a cell suspension with trypan blue dye, where viable cells exclude the dye and remain unstained, while non-viable cells take up the dye and appear blue under a light microscope. The mixture is then loaded onto a hemocytometer, and cells are counted to determine cell concentration and viability as follows: Cells/mL = (total cells counted/number of squares counted) × dilution factor × 10,000

Total cells in sample=cells/mL x total sample volume.

After 72 h of treatment with FANA-ASO, viable cells were again quantified, and cell counts were compared between groups to evaluate changes in growth.

### Real-time qPCR

Real-time qPCR was carried out using QuantiNova SYBR Green PCR Kit (Qiagen, Cat. No. 208054) according to the manufacturer’s instructions. Briefly, a total volume of 10 µL reaction mixture was prepared from 5 µL of 1x Master Mix, 1 µL of F and R primer (Table 2), 2 µL of cDNA (6 ng/µL), and 1 µL of RNase-free water. PCR cycling conditions: 95 °C for 2 min followed by 40 cycles of 95 °C for 5 s, and 61-63 °C for 30 s. All reactions were performed in duplicate. *GAPDH* was employed as an internal control. The 2^−ΔΔCT^ formula was used to calculate changes in expression level.

**TABLE 2:** Primer sequences.

Gene	Primers	Primer sequences (5′-3′)	Gene entry
*TIMAP*	TIMAP-F	GCC​GCA​AGA​AAG​TGT​CCT​TC	NM_015568.4
	TIMAP-R	ACA​AAT​CAG​GGC​TGA​CCT​TAT​TC	
*GAPDH*	GAPDH-F	GGA​GCG​AGA​TCC​CTC​CAA​AAT	NM_001357943.2
	GAPDH-R	GGC​TGT​TGT​CAT​ACT​TCT​CAT​GG	
*AICDA*	AICDA-F	CGCATCCTTTTGCCCCTGT	NM_020661.4
	AICDA-R	ACAGAGAAGACTTGAAGGACTGT	
*PAK3*	PAK3-F	CGCTGTCTTGAGATGGATGTGG	NM_002578
	PAK3-R	CAGTCTTAGCGGCTGCTGTTCT	
*BCL-2*	BCL2-F	ATCGCCCTGTGGATGACTGAGT	NM_000633
	BCL2-R	GCCAGGAGAAATCAAACAGAGGC	

### Protein extraction and quantification

Total protein was extracted using M-PER^®^Mammalian Protein Extraction Reagent (Thermo Scientific™, Cat. No. 75801) supplemented with protease inhibitors mini tablets (Thermo Scientific™, Cat. No. A32953) according to the manufacturer’s instructions. Briefly, cells were centrifuged at 2500 g for 10 min at 4 °C, washed with 1x PBS, and the cell pellets were incubated for 20 min on ice with M-PER reagent (200 µL per 1*10^6^ cells). Afterward, the cells were centrifuged at 14000 g for 15 min at 4 °C, and the supernatants were transferred to new tubes and stored at −80 for further investigation. Protein concentration was determined using the Bicinchoninic acid (BCA) (Pierce™ BCA) kit (Thermo Scientific™, Cat. No. 23225) according to the kit’s instructions. TIMAP protein levels were quantified using a human PPP1R16B sandwich ELISA Kit (ELK Biotechnology, Cat. No. ELK0855) according to the manufacturer’s instructions, and measurements were normalized based on the total protein concentration obtained from the BCA assay in corresponding samples.

### RNA sequencing and data analysis

The Qubit RNA assay was used to assess the quality of RNA samples before proceeding with RNA-seq. Azenta Biotech’s RNA sequencing service (Chelmsford, Massachusetts) was used to analyze all samples. Poly(A) selection method was applied before sequencing, and RNA sequencing was then conducted on the Illumina platform in a paired-end fashion with 2 × 150 bp reads. The RNA-seq data were received as Fastq files.

The FastQC and MultiQC tools (Galaxy Version 0.74), (RRID: SCR_014583) were used to evaluate the quality of reads [33–35]. The RNA-seq data were run through the Trim Galore tool (RRID: SCR_011847) to remove the adapter sequences from the reads before further analysis [36]. Following this, RNA-seq data were aligned to the human genome (GRCh38) using an ultrafast universal aligner RNA STAR tool (Galaxy Version 2.7.10b), (RRID: SCR_004463) [37]. The feature counts tool (Galaxy Version 2.0.3) was then applied to the alignment BAM output file to count RNA-seq reads [38]. After that, the Limma-Voom tool (Galaxy Version 3.50.1) was employed to identify the differentially expressed genes (DEGs) (adjusted p-value <0.05) between FANA -ASO- scramble treated and FANA -ASO- TIMAP treated samples [39, 40]. A volcano plot (Galaxy Version 0.0.5) and heatmap2 (Galaxy Version 3.1.3), (RRID:SCR_006281) were used to display the differentially expressed genes [34]. Furthermore, using the DAVID server (RRID:SCR_001881) and Enrichr tool (RRID:SCR_001575), we performed functional enrichment analysis to establish the gene categories and signaling pathways the differentially expressed genes belong to [41, 42].

### Statistical analysis

IBM SPSS Statistics 26 software (RRID:SCR_002865) was used to analyze the q-RT-PCR, ELISA, and cell counting data. Before performing statistical tests, a normality test was conducted to determine the distribution of the data. The unpaired, two-tailed Student’s t-test for independent samples, with equal variances assumed, was utilized for normally distributed data. At the same time, the Mann-Whitney U or Kruskal-Wallis test was used for non-normally distributed data. The data was presented as Mean ± SEM for normally distributed data and median with interquartile range (IQR) for non-normally distributed data. P-values of less than 0.05 were considered significant.

## Results

### TIMAP expression in B-cell NHL

**FIGURE 1:**
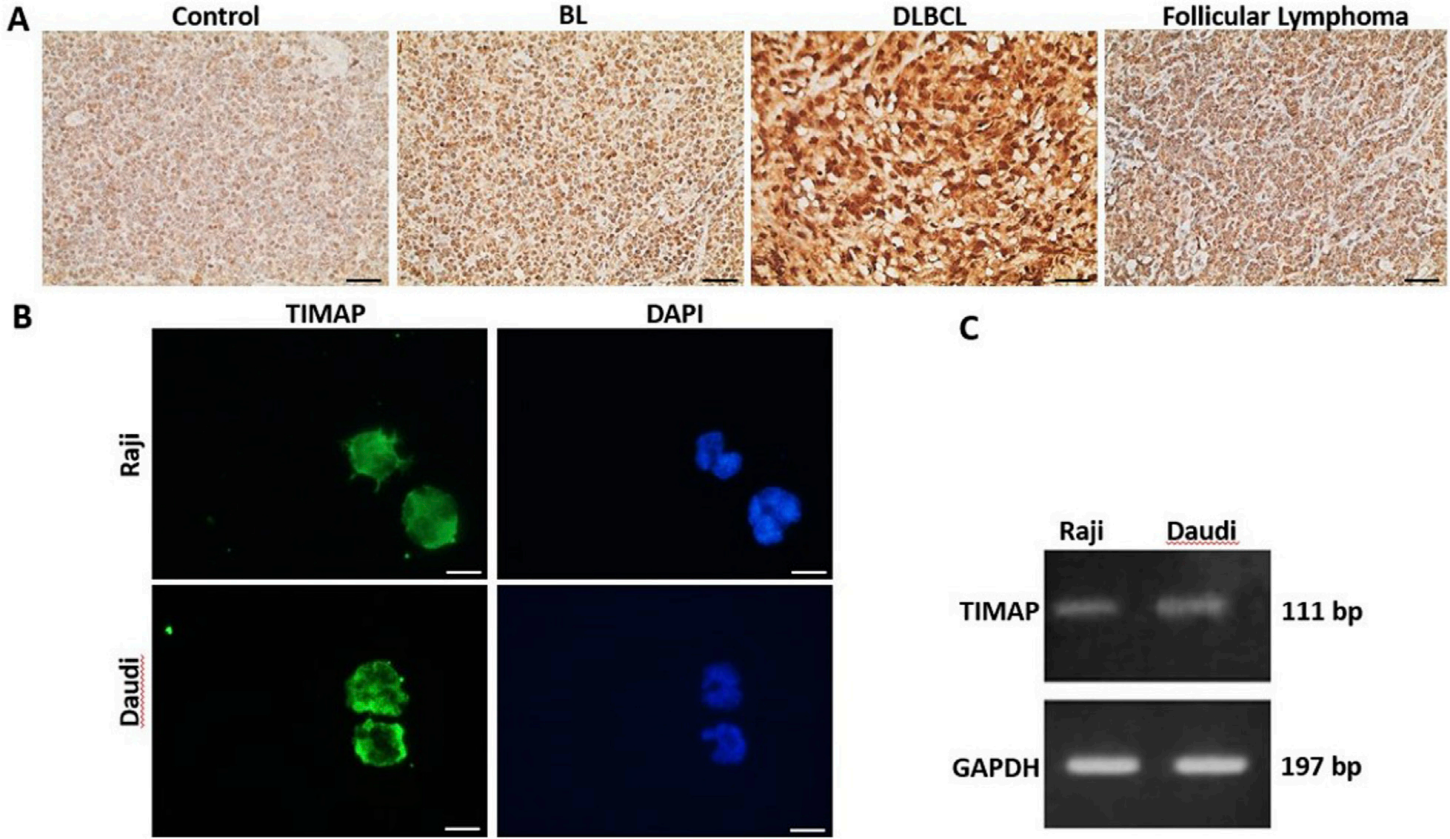
TIMAP expression in B-cell NHL. **(A)** IHC images captured at ×40 magnification of TIMAP expression in normal lymph node tissue (control), BL patient, DLBCL patient, and follicular lymphoma patient lymph node tissues. Scale bar 50µm. **(B)** Immunofluorescence images captured at ×100 magnification of TIMAP protein (green) in Raji and Daudi cell lines. Cell nuclei are depicted by DAPI staining (blue). Scale bar 50µm. **(C)** RT-PCR gel-electrophoresis demonstrates TIMAP and GAPDH expression in Raji and Daudi cell lines.

TIMAP protein expression was evaluated in lymph tissue sections from a healthy control, a BL patient, a DLBCL patient, and an FL patient by IHC. As shown in Figure 1A, TIMAP expression was upregulated in the lymphoma tissues compared to the normal lymph tissue. Notably, its expression was particularly high in DLBCL tissue. We next investigated TIMAP expression at both mRNA and protein levels in Raji and Daudi cell lines before knocking it down. Figures 1B,C show that TIMAP is expressed at both the protein and mRNA levels in Raji and Daudi cells, respectively.

### TIMAP knockdown analysis

**FIGURE 2:**
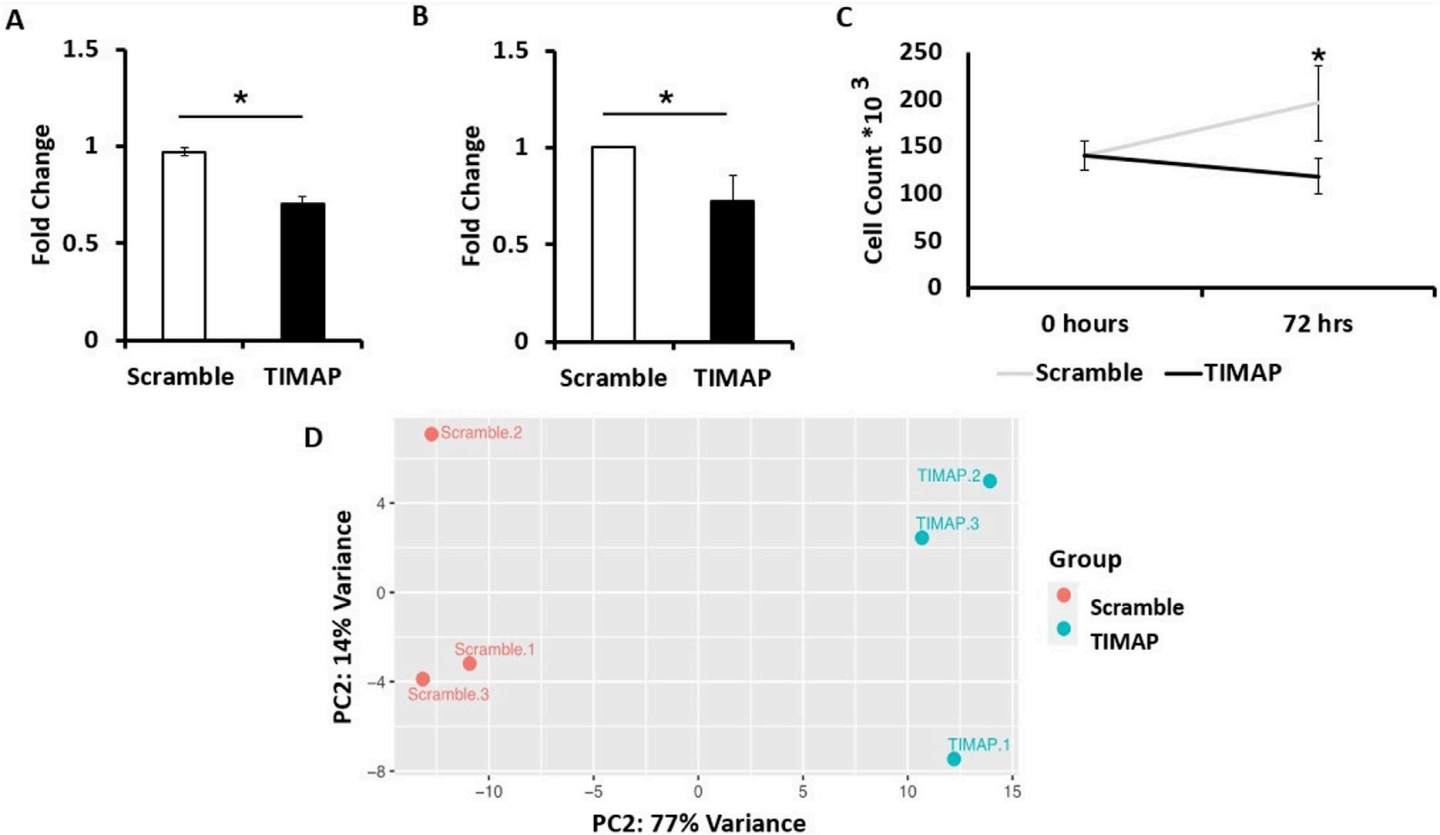
TIMAP Knockdown Analysis. **(A)** qPCR analysis of TIMAP mRNA expression (fold change) between FANA-ASO-scramble and FANA-ASO-TIMAP-treated cells. Data represent Mean ± SEM, * (*P* ≤ 0.05), n = 4 **(B)** ELISA analysis of TIMAP protein expression (fold change) between FANA-ASO-scramble and FANA-ASO-TIMAP-treated cells. Data represent Median ± IQR, * (*P* ≤ 0.05), n = 6. **(C)** Effect of TIMAP knockdown on cell growth. An equal number of Raji cells was seeded (0 h). After 72 h, viable cells were counted using trypan blue exclusion and a hemocytometer. TIMAP knockdown significantly reduced Raji cell growth compared with scramble-treated cells. Data represent Mean ± SEM. Statistical significance was determined using an independent-samples Kruskal-Wallis test, * (*P* ≤ 0.05), n = 9. **(D)** Principal component analysis (PCA) plot for FANA-ASO-TIMAP and FANA-ASO-Scramble treated samples. A two-dimensional PCA was conducted using normalized counts of genes between TIMAP and scramble samples. On the plot, each point represents a sample. The principal components (PC1, PC2) demonstrate the degree of variation between the groups.

TIMAP knockdown effectiveness was assessed at the mRNA and protein levels in the Raji cells after 72 h of FANA-ASO treatment using qPCR and ELISA, respectively. TIMAP mRNA was considerably decreased in cells treated with FANA-ASO-TIMAP (0.70 ± 0.04, *p* = 0.001, n = 4) compared to FANA-ASO-scramble control (0.97 ± 0.02) (Figure 2A), and TIMAP protein levels were decreased in FANA-ASO-TIMAP-treated cells (median = 0.73, IQR = 0.13, *p* = 0.002, n = 6) compared to FANA-ASO-scramble (median = 1.00, IQR = 0.00) (Figure 2B). These results indicate that FANA-ASO technology decreased TIMAP expression at the transcriptional and translational levels.

TIMAP knockdown has been previously shown to reduce endothelial cell growth [43]. Therefore, we further validated the efficiency of its knockdown by examining the impact on BL cell growth. An equal number of Raji cells was seeded and counted at baseline and after 72 h of transfection. While no significant difference was detected between scramble and TIMAP knockdown groups at baseline (*H* = 0.0, *df* = 1, *p* = 1.0), a Kruskal–Wallis test revealed a significant reduction in cell numbers following TIMAP knockdown compared with scramble-treated controls (*H* = 4.71, *df =* 1, *p* = 0.03) (Figure 2C). These findings indicate that TIMAP silencing impairs BL cell growth.

### Gene expression profile after TIMAP knockdown

To uncover the transcriptome profile after TIMAP knockdown, a paired-end Illumina RNA-seq was performed on FANA-ASO-scramble and FANA-ASO-TIMAP-treated cells from 3 independent experiments. In total, 39,470,217, 33,222,502, and 35,556,701 clean reads were obtained from three FANA-ASO-scramble-treated samples, while 43,443,071, 36,557,909, and 35,812,052 clean reads were obtained from three FANA-ASO-TIMAP-treated samples. All samples successfully mapped over 80% of reads to the current version of the human genome (GRCh38.p14) and met the quality standards required for downstream analysis.

Using the Ensembl annotation reference file, read counts were summarized at the gene level using featureCounts. A cutoff of 1 Count per Million (CPM) was used to select genes for differential expression analysis, resulting in 11,631 genes. Limma-Voom was then used to determine accurate DEGs based on the count tables generated from featureCounts. RNA-seq data were further evaluated through a Principal Component Analysis (PCA), which clustered samples with similar characteristics together (Figure 2D), indicating significant differences between control and knockdown samples.

**FIGURE 3:**
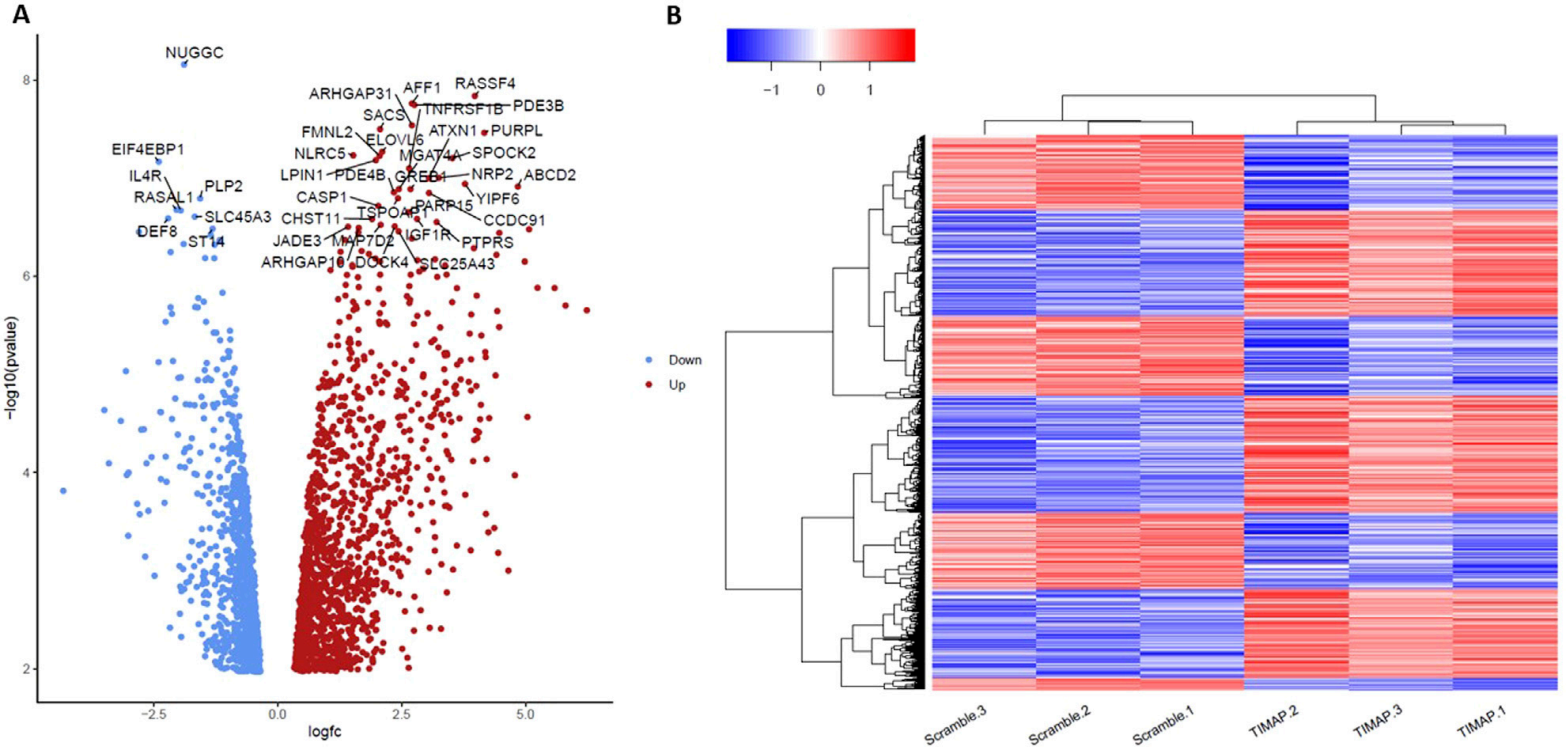
Evaluation of TIMAP Expression Patterns using Volcano plot and hierarchical clustering of DEGs. **(A)** A volcano plot showing all the genes with log2FC in TIMAP-knockdown and scramble samples on the X-axis, while the -log10 adjusted p-value is presented on the Y-axis. The grey dots indicate genes that are not statistically significant (using 0.05 of the adjusted p-value as a threshold), the red points represent genes that are considerably overexpressed (log2FC > 0), and the blue points represent significantly under-expressed genes (log2FC < 0). **(B)** A Heatmap of the normalized counts for the DEGs in Scramble 1-3 samples and TIMAP-knockdown 1–3 samples. The blue color indicates low expression of certain genes in the sample, while the red color indicates high expression of those genes. Hierarchical clustering by heatmap has successfully differentiated the DEGs between scramble and TIMAP knockdown samples.

A total of 2,368 genes were substantially dysregulated (adjusted *P* < 0.05 as the threshold), with 1,326 upregulated genes (log2FC > 0) and 1,042 downregulated genes (log2FC < 0). The distinct expression patterns in various samples were visualized by a volcano plot and hierarchical clustering, as illustrated in Figures 3A,B, respectively. The top 50 differentially up-and down-regulated genes are summarized in Tables 3, 4, respectively.

**TABLE 3:** Top 50 up-regulated DEGs after TIMAP knockdown.

GeneID	Gene name	Description	Feature	Log2FC	Adj.P.Val
ENSG00000077264	*PAK3*	p21 (RAC1) activated kinase 3	protein coding	6.23699785	0.00025039
ENSG00000186297	*GABRA5*	gamma-aminobutyric acid type A receptor subunit alpha5	protein coding	5.807702811	0.000240473
ENSG00000163362	*INAVA*	innate immunity activator	protein coding	5.587054181	0.000189518
ENSG00000172296	*SPTLC3*	serine palmitoyl transferase long chain base subunit 3	protein coding	5.242882496	0.000189518
ENSG00000152128	*TMEM163*	transmembrane protein 163	protein coding	5.039945964	0.001050781
ENSG00000099282	*TSPAN15*	tetraspanin 15	protein coding	4.985660189	0.000131879
ENSG00000173208	*ABCD2*	ATP binding cassette subfamily D member 2	protein coding	4.846412503	7.51E-05
ENSG00000257261	*SLC38A4-AS1*	SLC38A4 Antisense RNA 1	lncRNA	4.655443681	0.010921537
ENSG00000104177	*MYEF2*	myelin expression factor 2	protein coding	4.47245795	9.81E-05
ENSG00000235831	*BHLHE40-AS1*	BHLHE40 antisense RNA 1	lncRNA	4.470596306	0.000318507
ENSG00000111249	*CUX2*	cut like homeobox 2	protein coding	4.450363672	0.008324052
ENSG00000092051	*JPH4*	junctophilin 4	protein coding	4.431012223	0.000252779
ENSG00000204161	*TMEM273*	transmembrane protein 273	protein coding	4.414377442	0.000128125
ENSG00000137491	*SLCO2B1*	solute carrier organic anion transporter family member 2B1	protein coding	4.397003908	0.00064369
ENSG00000142347	*MYO1F*	myosin IF	protein coding	4.371096523	0.005564985
ENSG00000250358	*LINC02200*	long intergenic non-protein coding RNA 2200	lncRNA	4.268124613	0.001068858
ENSG00000078018	*MAP2*	microtubule associated protein 2	protein coding	4.251541511	0.005947381
ENSG00000071909	*MYO3B*	myosin IIIB	protein coding	4.216609343	0.001940389
ENSG00000215386	*MIR99AHG*	mir-99a-let-7c cluster host gene	lncRNA	4.201310683	0.004459451
ENSG00000198216	*CACNA1E*	calcium voltage-gated channel subunit alpha1 E	protein coding	4.197890373	0.000503653
ENSG00000162714	*ZNF496*	zinc finger protein 496	protein coding	4.189331705	0.00047376
ENSG00000250337	*PURPL*	p53 upregulated regulator of p53 levels	lncRNA	4.169379922	5.75E-05
ENSG00000146950	*SHROOM2*	shroom family member 2	protein coding	4.110414976	0.000366862
ENSG00000114646	*CSPG5*	chondroitin sulfate proteoglycan 5	protein coding	4.100209392	0.00087828
ENSG00000113946	*CLDN16*	claudin 16	protein coding	4.084168866	0.001070074
ENSG00000165868	*HSPA12A*	heat shock protein family A (Hsp70) member 12A	protein coding	4.060589474	0.00127637
ENSG00000276231	*PIK3R6*	phosphoinositide-3-kinase regulatory subunit 6	protein coding	4.026197817	0.001413494
ENSG00000136531	*SCN2A*	sodium voltage-gated channel alpha subunit 2	protein coding	4.008373053	0.000704814
ENSG00000179088	*C12orf42*	chromosome 12 open reading frame 42	protein coding	4.005637675	0.001250976
ENSG00000107551	*RASSF4*	Ras association domain family member 4	protein coding	3.978025743	5.24E-05
ENSG00000163518	*FCRL4*	Fc receptor like 4	protein coding	3.964185129	0.00145664
ENSG00000101255	*TRIB3*	tribbles pseudo kinase 3	protein coding	3.958941693	0.000120615
ENSG00000198933	*TBKBP1*	TBK1 binding protein 1	protein coding	3.94411888	0.00198347
ENSG00000173198	*CYSLTR1*	cysteinyl leukotriene receptor 1	protein coding	3.905841489	0.0002639
ENSG00000188487	*INSC*	INSC spindle orientation adaptor protein	protein coding	3.902938238	0.000518891
ENSG00000075651	*PLD1*	phospholipase D1	protein coding	3.901750708	0.000751531
ENSG00000171016	*PYGO1*	pygopus family PHD finger 1	protein coding	3.895986603	0.008004992
ENSG00000135821	*GLUL*	glutamate-ammonia ligase	protein coding	3.87401119	0.000354305
ENSG00000154102	*C16orf74*	chromosome 16 open reading frame 74	protein coding	3.855659004	0.000679729
ENSG00000116833	*NR5A2*	nuclear receptor subfamily 5 group A member 2	protein coding	3.785153504	0.003323437
ENSG00000181704	*YIPF6*	Yip1 domain family member 6	protein coding	3.781298376	7.51E-05
ENSG00000149403	*GRIK4*	glutamate ionotropic receptor kainate type subunit 4	protein coding	3.693682798	0.000671969
ENSG00000064225	*ST3GAL6*	ST3 beta-galactoside alpha-2,3-sialyltransferase 6	protein coding	3.690727682	0.000522351
ENSG00000138771	*SHROOM3*	shroom family member 3	protein coding	3.668740688	0.001068858
ENSG00000124570	*SERPINB6*	serpin family B member 6	protein coding	3.623297993	0.000552965
ENSG00000237372	*LINC03062*	long intergenic non-protein coding RNA 3062	lncRNA	3.617369981	0.000189518
ENSG00000283526	*PRRT1B*	proline rich transmembrane protein 1B	protein coding	3.615650355	0.007432053
ENSG00000162654	*GBP4*	guanylate binding protein 4	protein coding	3.596555527	0.000701994
ENSG00000072840	*EVC*	EvC ciliary complex subunit 1	protein coding	3.584536839	0.000775413
ENSG00000163554	*SPTA1*	spectrin alpha, erythrocytic 1	protein coding	3.539517179	0.010756775

**TABLE 4:** Top 50 down-regulated DEGs after TIMAP knockdown.

GeneID	Gene name	Description	Feature	Log2FC	adj.P.Val
ENSG00000205837	*LINC00487*	long intergenic non-protein coding RNA 487	lncRNA	−4.320875734	0.003139371
ENSG00000188783	*PRELP*	proline and arginine rich end leucine rich repeat protein	protein coding	−3.493418349	0.000951158
ENSG00000162105	*SHANK2*	SH3 and multiple ankyrin repeat domains 2	protein coding	−3.401540724	0.002057955
ENSG00000130487	*KLHDC7B*	kelch domain containing 7B	protein coding	−3.160369721	0.00109221
ENSG00000143995	*MEIS1*	Meis homeobox 1	protein coding	−3.058968491	0.000604104
ENSG00000164694	*FNDC1*	fibronectin type III domain containing 1	protein coding	−3.041008069	0.002498708
ENSG00000163884	*KLF15*	KLF transcription factor 15	protein coding	−3.010938764	0.006295845
ENSG00000165457	*FOLR2*	folate receptor beta	protein coding	−3.003218524	0.002418629
ENSG00000203710	*CR1*	complement C3b/C4b receptor 1 (Knops blood group)	protein coding	−2.829643321	0.003776015
ENSG00000102098	*SCML2*	Scmpolycomb group protein like 2	protein coding	−2.795587703	9.81E-05
ENSG00000168491	*CCDC110*	coiled-coil domain containing 110	protein coding	−2.750088398	0.001241616
ENSG00000273018	*FAM106A*	family with sequence similarity 106 member A	lncRNA	−2.695369633	0.001241616
ENSG00000254030	*IGLC5*	immunoglobulin lambda constant 5 (pseudogene)	IG_C_pseudogene	−2.669444649	0.008864195
ENSG00000078114	*NEBL*	nebulette	protein coding	−2.605029011	0.00426347
ENSG00000211898	*IGHD*	immunoglobulin heavy constant delta	IG_C_gene	−2.479821045	0.011755158
ENSG00000182866	*LCK*	LCK proto-oncogene, Src family tyrosine kinase	protein coding	−2.398245479	0.000533898
ENSG00000187840	*EIF4EBP1*	eukaryotic translation initiation factor 4E binding protein1	protein coding	−2.395071203	6.10E-05
ENSG00000182168	*UNC5C*	unc-5 netrin receptor C	protein coding	−2.364945932	0.000986908
ENSG00000270959	*LPP-AS2*	LPP antisense RNA 2	lncRNA	−2.353260745	0.00262887
ENSG00000117020	*AKT3*	AKT serine/threonine kinase 3	protein coding	−2.350106042	0.000988183
ENSG00000224187	*LINC01991*	Long Intergenic Non-Protein Coding RNA 1991	lncRNA	−2.257073659	0.000295627
ENSG00000230426	*LINC01036*	long intergenic non-protein coding RNA 1036	lncRNA	−2.255122831	0.00187507
ENSG00000167995	*BEST1*	bestrophin 1	protein coding	−2.238896365	0.002732034
ENSG00000140995	*DEF8*	differentially expressed in FDCP 8 homolog	protein coding	−2.206241878	9.52E-05
ENSG00000125888	*BANF2*	BANF family member 2	protein coding	−2.168635127	0.025987585
ENSG00000082458	*DLG3*	discs large MAGUK scaffold protein 3	protein coding	−2.156337647	0.000124827
ENSG00000182963	*GJC1*	gap junction protein gamma 1	protein coding	−2.130493525	0.000261897
ENSG00000160505	*NLRP4*	NLR family pyrin domain containing 4	protein coding	−2.129066591	0.001070074
ENSG00000179750	*APOBEC3B*	apolipoprotein B mRNA editing enzyme catalytic subunit3B	protein coding	−2.10871196	0.000523548
ENSG00000167483	*NIBAN3*	niban apoptosis regulator 3	protein coding	−2.063615734	0.000814506
ENSG00000111344	*RASAL1*	RAS protein activator like 1	protein coding	−2.039040937	9.22E-05
ENSG00000146215	*CRIP3*	cysteine rich protein 3	protein coding	−2.010201596	0.001326338
ENSG00000132464	*ENAM*	enamelin	protein coding	−1.976291645	0.010163259
ENSG00000246705	*H2AJ*	H2A.J histone	protein coding	−1.969529943	0.020995928
ENSG00000151322	*NPAS3*	neuronal PAS domain protein 3	protein coding	−1.966339059	0.002197977
ENSG00000077238	*IL4R*	interleukin 4 receptor	protein coding	−1.952740272	9.22E-05
ENSG00000107331	*ABCA2*	ATP binding cassette subfamily A member 2	protein coding	−1.950283708	0.000658604
ENSG00000185189	*NRBP2*	nuclear receptor binding protein 2	protein coding	−1.943895447	0.030072386
ENSG00000144331	*ZNF385B*	zinc finger protein 385B	protein coding	−1.904057567	0.010662957
ENSG00000145012	*LPP*	LIM domain containing preferred translocation partner in lipoma	protein coding	−1.893440473	0.000113202
ENSG00000232265	*LINC02805*	long intergenic non-protein coding RNA 2805	lncRNA	−1.886336509	0.001762078
ENSG00000189233	*NUGGC*	nuclear GTPase, germinal center associated	protein coding	−1.885608864	5.24E-05
ENSG00000116157	*GPX7*	glutathione peroxidase 7	protein coding	−1.881730165	0.014259455
ENSG00000171766	*GATM*	glycine amidino transferase	protein coding	−1.85258952	0.001675188
ENSG00000148175	*STOM*	stomatin	protein coding	−1.851974907	0.00087828
ENSG00000111732	*AICDA*	activation induced cytidine deaminase	protein coding	−1.83973866	0.013005594
ENSG00000212123	*PRR22*	proline rich 22	protein coding	−1.78860534	0.012811673
ENSG00000147437	*GNRH1*	gonadotropin releasing hormone 1	protein coding	−1.780287627	0.008879193
ENSG00000132744	*ACY3*	amino acylase 3	protein coding	−1.769829408	0.001632406
ENSG00000012779	*ALOX5*	arachidonate 5-lipoxygenase	protein coding	−1.768707059	0.00087828

### Validation of key DEGs by qPCR

**FIGURE 4:**
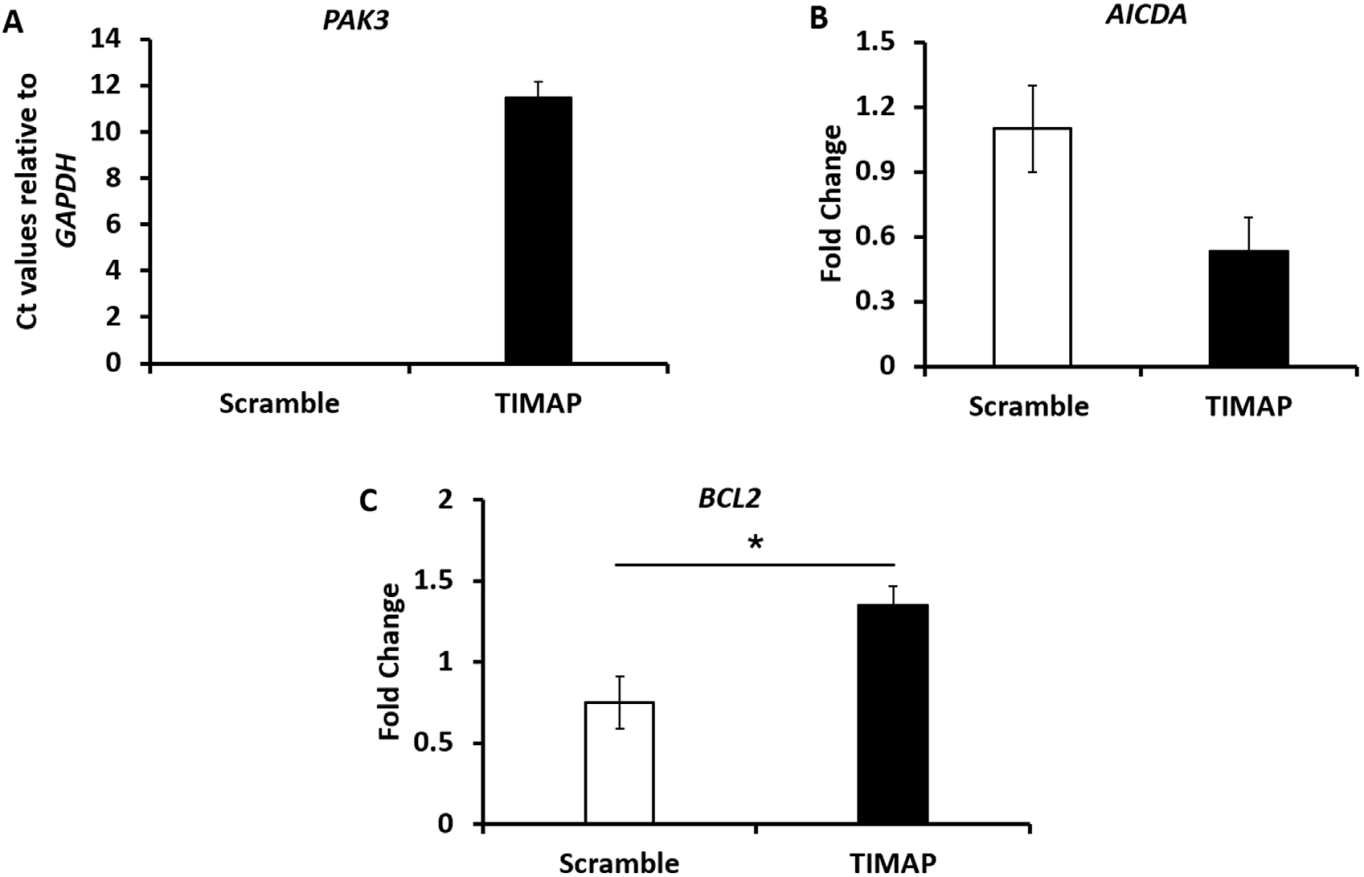
Validation of selected DEGs by qPCR. **(A)** qPCR analysis of *PAK3* mRNA expression between FANA-ASO-scramble and FANA-ASO-TIMAP-treated cells, as represented by the mean normalized Ct value relative to *GAPDH*, n = 3. **(B,C)** qPCR analysis of *AICDA* and *BCL2* mRNA expression (fold change) in FANA-ASO-scramble and FANA-ASO-TIMAP-treated cells. Data represent mean ± SEM of three independent experiments, * (*P* ≤ 0.05).

To validate key DEGs relevant to BL pathogenesis, qPCR was performed for *PAK3*, *AICDA*, and *BCL-2*. As shown in Figure 4A, *PAK3* expression was absent in FANA-ASO-Scramble–treated cells but induced upon TIMAP knockdown; this expression was also undetectable at baseline in Raji BL cells (data not shown). Because *PAK3* was not measurable in the scramble control, expression is shown as normalized Ct values relative to *GAPDH* in knockdown samples. Figure 4B demonstrates that *AICDA* expression was downregulated in FANA-ASO-TIMAP–treated cells (0.53 ± 0.16, *P* = 0.07, n = 4) compared to the scramble control (1.1 ± 0.2). While TIMAP knockdown induced *BCL-2* expression (1.35 ± 0.12, *P* = 0.03, n = 4) compared to scramble (0.75 ± 0.16), as shown in Figure 4C. These results are consistent with the RNA-seq analysis and confirm that even partial TIMAP silencing alters the expression of genes central to BL pathogenesis.

### Gene ontology (GO) analysis of DEGs

**FIGURE 5:**
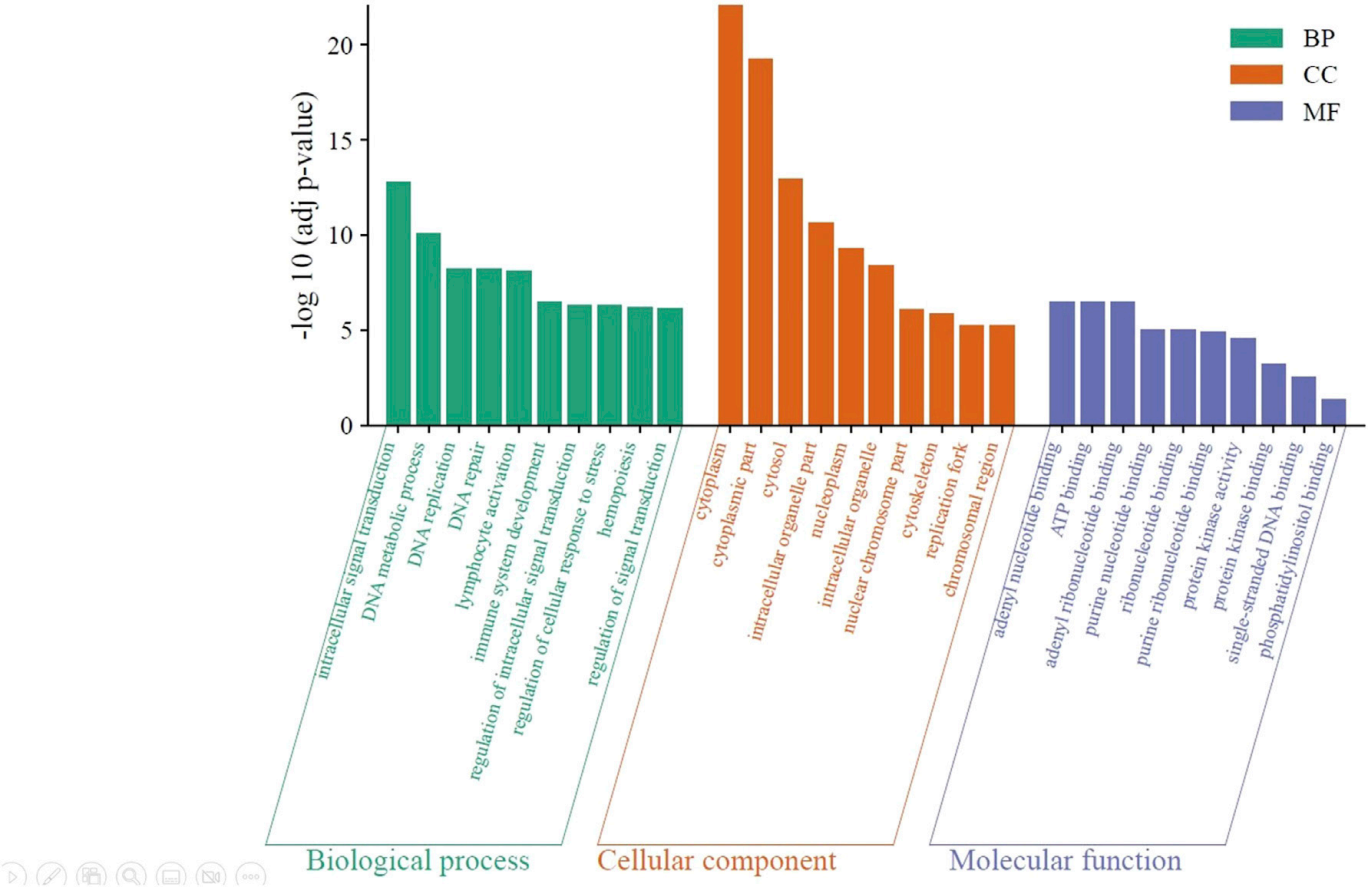
Significantly enriched GO terms in the identified DEGs. The green bars represent the top 10 biological processes, the orange bars represent the top 10 cellular components, and the violet bars represent the top 10 molecular functions. BP: biological processes; CC: cellular component; and MF: molecular function.

Using the DAVID server, the molecular functions (MF), biological processes (BP), and cellular components (CC) of the DEGs were clustered to reveal the significantly enriched GO terms (adjusted *p* < 0.05) (Figure 5). According to the results, biological processes are primarily involved in the positive regulation of cellular processes (GO: 0048522), cell communication (GO: 0010646), intracellular signal transduction (GO: 0035556), DNA metabolic process (GO: 0006259), and leukocyte activation (GO: 0045321). Cellular components include the cytoplasm (GO: 0005737) and cytosol (GO: 0005829), intracellular organelles (GO: 0043229), nucleoplasm (GO: 0005654), and cytoskeleton (GO: 0005856). Among the molecular functions of DEGs are kinase activity (GO: 0016301) and kinase binding (GO: 0019900), phosphor-transferase activity; alcohol group as acceptor (GO: 0016773), nucleotide binding (GO: 0000166), phospholipid binding (GO: 0005543), and phosphatidylinositol binding (GO: 0035091).

### KEGG analysis of DEGs

**FIGURE 6:**
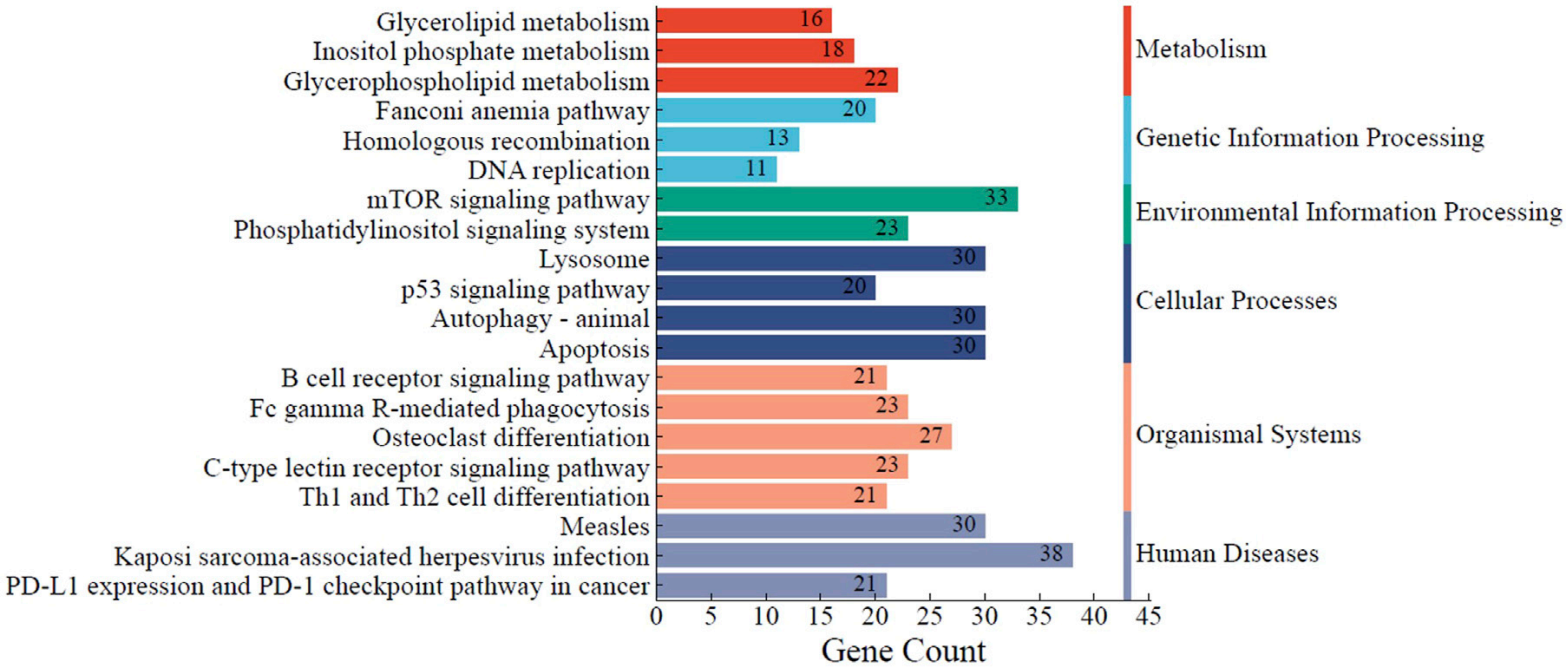
KEGG pathway analysis of the identified DEGs. The top 20 significantly enriched KEGG pathways to which DEGs belong are summarized. The functional KEGG pathways and the number of DEGs in each pathway are represented by the Y- and X-axes, respectively. * KEGG, Kyoto Encyclopedia of Genes and Genomes.

Based on the KEGG pathway database, the Enrichr tool was used to find the most significantly enriched pathways to which the DEGs belong. The findings in Figure 6 demonstrate that DEGs were clustered in several significant KEGG pathways (adjusted *p* < 0.05), including the p53 signaling pathway (hsa04115), apoptosis (hsa04210), B-cell receptor (BCR) signaling pathway (hsa04662), homologous recombination (hsa03440), mTOR signaling pathway (hsa04150), and DNA replication (hsa03030).

## Discussion

TIMAP is highly expressed in several solid tumors and blood cancer cells, including BL [23]. However, its molecular function in cancer has not been fully investigated. Many previous studies convincingly demonstrated TIMAP’s role in regulating various cellular processes that are known to be implicated in tumor pathogenesis through its interaction with key complicit molecules [20]. Among those are several nuclear proteins involved in the RNA splicing mechanism, such as U5 snRNP, SFPQ, and hnRNPA1 [27, 29], indicating a possible function of TIMAP in transcriptional regulation. In this study, RNA-seq was conducted to identify the transcriptome profile of BL cells after TIMAP silencing. Our analysis revealed 2,368 DEGs and 20 signaling pathways dysregulated in BL.

RNA-seq is one of the most sensitive and widely used methods for observing how cells respond to treatment and eventually identifying the dysregulated genes and pathways [44, 45]. In this first-of-its-kind work, RNA-seq was performed on Raji BL cells treated with FANA-ASO-TIMAP, and the gene expression profile was compared to that of the cells treated with FANA-ASO-Scramble control to discover TIMAP-responsive genes. The PCA plot highlighted considerable similarities between the TIMAP knockdown samples and distinguished them from the control. A total of 2,368 genes were found to be differentially expressed in response to TIMAP downregulation, of which 1,326 were upregulated and 1,042 were downregulated. On the heatmap, the clustering of those DEGs provided consistent expression patterns for each treatment group, further confirming the distinguished transcriptome profile of TIMAP knockdown cells.

In our study, 2 µM FANA-ASO was used to target TIMAP in BL cells, achieving approximately 30% knockdown efficiency after 72 h. This result is notably lower than an earlier study, which used 8 µM FANA-ASO to knock down KCTD15 in B-cell leukemia cells, resulting in a much higher 80% knockdown efficiency on days 8–16 [31]. Additionally, previous research on silencing ABI1 in healthy CD34^+^ cells using FANA-ASO achieved approximately 40% knockdown after 48 h of treatment [46]. These discrepancies may be attributed to differences in FANA-ASO concentration, treatment duration, cell type, and the target gene.

Despite achieving only a 30% knockdown of TIMAP, our study was still able to identify a significant number of DEGs with deregulated expression. This indicates that even partial silencing of TIMAP can lead to substantial alterations in the transcriptome of BL cells. The identified DEGs were associated with critical pathways involved in cell survival, proliferation, and apoptosis. Consistently, TIMAP knockdown attenuated cell growth, in line with previous findings [43] and the predicted impact on growth signaling pathways identified here. These results suggest that even a modest knockdown of TIMAP can have profound effects on the cellular processes driving tumorigenesis. Identifying key genes and pathways affected by TIMAP silencing provides valuable insights into potential therapeutic targets, despite the limited knockdown efficiency. Hence, future research that utilizes variable gene manipulation techniques is vital to elucidate the precise mechanisms through which TIMAP regulates these molecules and pathways in BL.

In addition to the transcriptomic alterations observed upon TIMAP silencing, our IHC and cell line data provide important evidence that TIMAP is upregulated in B-cell non-Hodgkin lymphomas. Specifically, TIMAP protein expression was markedly elevated in BL, DLBCL, and FL tissues compared to normal lymph tissue, with particularly strong expression in DLBCL. Notably, *TIMAP* transcript was also among the upregulated genes in a previous transcriptomic profiling study of DLBCL [25], providing independent support for our findings. Consistently, both Raji and Daudi BL cell lines exhibited detectable TIMAP expression at the mRNA and protein levels. These findings suggest that TIMAP overexpression may represent a common feature across multiple B-cell lymphoma subtypes, rather than being restricted to BL. This is consistent with its known role in regulating pathways central to cell survival and proliferation, including PI3K/Akt/mTOR [43]. The observation of particularly high TIMAP expression in DLBCL further raises the possibility that TIMAP may contribute to disease aggressiveness or heterogeneity in B-cell lymphomas. Future studies involving patient cohorts and subtype-specific analyses are necessary to clarify whether TIMAP expression has prognostic significance and to determine its potential as a biomarker or therapeutic target across B-cell malignancies.

Among the most upregulated genes in our study was *PAK3*, a member of the PAK family of serine/threonine kinases originally identified as downstream effectors of the Rho GTPases Cdc42 and Rac [47]. PAKs are divided into two groups based on the sequence and structure: Group I PAKs (PAK1, PAK2, and PAK3) and Group II PAKs (PAK4, PAK5, and PAK6) [48]. PAKs regulate various cellular processes that are often disrupted in cancer, including cell survival, cell growth, and cytoskeleton remodeling [49]. PAKs are frequently upregulated in various tumors and influence several oncogenic signaling pathways that promote resistance to apoptosis, uncontrolled cell proliferation, and drug resistance, making them potential targets for therapy [48, 50]. Consequently, PAK inhibitors have recently been examined for their therapeutic activity in several cancers, including lymphoma [48, 50–52].

Previous studies have revealed a negative impact of high PAK1 and PAK2 expression on relapse-free survival in T-cell lymphoblastic lymphoma (T-LBL) patients, and PAK inhibitors were shown to attenuate T-LBL growth and enhance chemosensitivity to doxorubicin [48, 50]. To date, the role of PAK3 in cancer remains elusive. It is upregulated in head and neck squamous cell carcinoma and is considered a prognostic marker in glioblastoma multiforme [23]. However, it is best known for its biological function in the nervous system, where it is predominantly expressed and plays an important role in synaptic plasticity [53]. Since TIMAP is also predominantly expressed in the nervous system [21, 23] and is a prognostic marker in glioblastoma multiform [27] and head and neck cancer [23, 26], it is plausible that it might be associated with PAK3 regulation. Interestingly, our qPCR analysis revealed that while PAK3 expression was undetectable in FANA-ASO-scramble–treated cells and in untreated cells, it was induced upon TIMAP knockdown. Hence, our findings suggest the importance of the TIMAP-PAK3 axis in BL, which prompts future investigations to address this relationship.

In contrast to *PAK3*, *LINC00487*, a long intergenic non-coding RNA (lncRNA), was the most downregulated gene in our study. *LINC00487* is one of the core genes in the germinal center B cells, contributing to B-cell development [54] and a key gene for predicting prognosis in DLBCL [55]. Research has shown that LINC00487 and other lncRNAs are linked to the enzyme activation-induced cytidine deaminase (AICDA), also known as AID [56]. AICDA is a DNA-modifying enzyme and one of the key genes in germinal center B cells [54, 57]. It plays a crucial role in generating diversity in immunoglobulins by converting cytosine to uracil in the variable and switch regions of immunoglobulin genes. This process leads to C: G mismatches, promoting class switching from IgM and IgD to other isotypes [57].

AICDA has been identified as an oncogene due to its ability to modify DNA, which enhances chromosomal translocations between the *c-myc* oncogene and immunoglobulin genes, induces point mutations in oncogenes, alters DNA methylation, and activates translocations of non-immunoglobulin genes [57–62]. It is also overexpressed in DLBCL [63] where TIMAP showed the most upregulation in our study. A recent study suggests that upregulation of AICDA significantly promotes cell proliferation, migration, genomic instability, and resistance to chemotherapy in B-cell lymphoma, indicating that AICDA could be a potential therapeutic target [64]. Furthermore, AICDA was identified as a driver of epigenetic heterogeneity in B-cell lymphoma, and its overexpression aggravates the disease [65].

Interestingly, AICDA was also downregulated in our study, suggesting that reduced TIMAP expression negatively impacts AICDA transcription. This may, in turn, decrease its effect on BL cell growth. This finding is consistent with previous reports that TIMAP downregulation adversely affects cell growth [43, 66], as well as with the reduced cell growth observed in FANA-ASO-TIMAP-treated cells in our study. Moreover, since *AICDA* and *LINC00487* are predominantly expressed in the germinal center B-cells, which are the precursors of BL, and downregulated in BL cells by TIMAP knockdown in the current study, this suggests that they might be associated with TIMAP in regulating normal and cancer B-cell development.

Another upregulated gene in our study involved in cell survival regulation is the p53 upregulated regulator of p53 levels (*PURPL*). Normally, P53 activation drives cells with translocations to undergo apoptosis [67]. However, P53 is inhibited in numerous types of cancer, resulting in the survival of aberrant cells that eventually develop into malignant cells [68–70]. PURPL has been shown to deplete P53 levels in colorectal cancer cells through its interaction with the Myb-binding protein 1A (MYBBP1A), a protein that binds to and stabilizes p53, and PURPL-deficient cells exhibit impaired tumor growth [71]. Furthermore, PURPL is overexpressed in gastric cancer, where it promotes cell growth, migration, survival, and invasion [72]. Therefore, our findings indicate a potential regulatory role of TIMAP in the p53 pathway.

An important gene that was also found to be upregulated in our RNA-seq and validated by qPCR is *BCL2*, a central regulator of apoptosis in B cells. BCL2 is an anti-apoptotic protein that promotes cell survival by inhibiting mitochondrial outer membrane permeabilization, thereby blocking caspase activation [73]. Dysregulated expression of BCL2 is a hallmark of several B-cell malignancies, including FL and DLBCL, where it often arises from the characteristic t(14; 18) (q32; q21) translocation [74]. Although BL is classically considered BCL2-negative, subsets of BL can display BCL2 expression [75], which may contribute to treatment resistance and disease heterogeneity. Raji cells, on the other hand, express BCL2 [76], which is consistent with our results. *BCL2* was not among the top 50 upregulated genes in our RNA-seq analysis. Still, we validated its expression due to its well-established critical role in B-cell lymphoma biology, especially since TIMAP protein expression was upregulated in different B-cell lymphoma cases in the current study, suggesting it might play a common role in these malignancies. The induction of *BCL2* expression following TIMAP knockdown in our study implies a potential compensatory mechanism by which BL cells may counterbalance the pro-apoptotic stress induced by reduced TIMAP expression. This aligns with our observed reduction in cell growth, indicating that despite *BCL2* upregulation, TIMAP silencing may override survival signals and shift the balance toward apoptosis. These findings raise the possibility that TIMAP may be indirectly linked to apoptotic regulation through its influence on *BCL2* expression, a hypothesis that warrants further mechanistic investigation.

Through GO analysis using the DAVID server, the DEGs revealed by TIMAP knockdown were categorized into three categories (biological processes (BP), cellular components (CC), and molecular functions (MF)). DEGs were associated with various GO terms, including signal transduction via intracellular signaling cascades, cytoskeleton organization, apoptosis, replication, repair, hemopoiesis, leukocyte activation, cell communication regulation, and kinase activity. These pathways are consistent with previously published research on TIMAP [20]. Ultimately, prospective research dissecting these significantly enriched GO terms could help us precisely understand how TIMAP is implicated in B-cell lymphomagenesis.

Among the signaling pathways impacted by TIMAP silencing in our study are mTOR and BCR. The survival of malignant B cells in BL depends on the tonic BCR signaling pathway, as evidenced by the death of BL cell lines when BCR components are knocked down [77, 78]. BCR activates PI3K/Akt/mTOR signaling pathways to overcome the pro-apoptotic effect of *c-myc* overexpression and stimulate B cell proliferation; thus, inhibition of PI3K and mTOR pathways was associated with BL cell death and increased sensitivity to chemotherapy [79–81]. In support of our findings, Obeidat et al reported that TIMAP downregulation inhibits cellular proliferation and survival in EC by attenuating the PI3K/Akt signaling pathway [43]. Further research is recommended to expand TIMAP’s molecular mechanism regarding these pathways in the context of B-cell lymphoma pathogenesis. This may, eventually, facilitate its incorporation into diagnostic and therapeutic approaches.

While our study focused on BL, TIMAP has been reported to be expressed in several solid tumors [23, 24, 26]. Extending transcriptomic profiling of TIMAP knockdown to other malignancies could uncover both shared and cancer–type–specific targets. Such comparative analyses may reveal whether TIMAP regulates universal oncogenic pathways or acts through lineage-restricted mechanisms.

## Conclusion

This study highlights TIMAP as a potential therapeutic target in B-cell lymphoma by demonstrating that even partial silencing in Raji BL cells induces profound transcriptomic and phenotypic changes. TIMAP suppression altered the gene expression of key regulators, including *PAK3*, *AICDA, and BCL2*, and impacted critical pathways such as BCR and PI3K/Akt/mTOR, confirming its role in cell growth. Although limited by the lack of siRNA or single-oligonucleotide validation, our findings establish a foundation for future work to dissect the TIMAP–PAK3, TIMAP–BCL2, and TIMAP–AICDA axes and to evaluate the functional impact of TIMAP silencing *in vivo*. Importantly, TIMAP overexpression in BL, DLBCL, and FL tissues suggests a broader role in B-cell lymphomagenesis, which prompts the investigation of its clinical significance as a prognostic marker and therapeutic target across lymphoma subtypes.

## Data Availability

The datasets presented in this study can be found in online repositories. The names of the repository/repositories and accession number(s) can be found below: https://www.ncbi.nlm.nih.gov/, PRJNA1172000.
